# Chronic disease management perspectives of colorectal cancer survivors using the Veterans Affairs healthcare system: a qualitative analysis

**DOI:** 10.1186/s12913-018-2975-3

**Published:** 2018-03-09

**Authors:** Leah L. Zullig, Karen M. Goldstein, Hayden B. Bosworth, Sara M. Andrews, Susanne Danus, George L. Jackson, Dawn Provenzale, Morris Weinberger, Michael J. Kelley, Corrine I. Voils

**Affiliations:** 1 Center for Health Services Research in Primary Care, Durham Veterans Affairs Health Care System, 411 West Chapel Hill Street, Suite 600, Durham, NC 27701 USA; 2 Department of Population Health Sciences, Duke University Medical Center, Durham, USA; 3 Department of Medicine, Duke University Medical Center, Durham, USA; 4 Department of Psychiatry and Behavioral Sciences and School of Nursing, Duke University, Durham, USA; 5 Cooperative Studies Program Epidemiology Center-Durham, Durham, NC USA; 6 Department of Health Policy and Management, University of North Carolina, Chapel Hill, USA; 7 Department of Veterans Affairs, Washington, DC USA; 8 Hematology-Oncology Service, Durham Veterans Affairs Health Care System, Durham, USA; 9 William S. Middleton Memorial Veterans Hospital, Madison, USA; 10 Department of Surgery, University of Wisconsin School of Medicine and Public Health, Madison, USA

**Keywords:** Veteran health, Colorectal cancer, Cancer survivorship, Qualitative research, Chronic disease, Cardiovascular disease, Medication adherence

## Abstract

**Background:**

Colorectal cancer (CRC) is the third most commonly diagnosed cancer in the US. CRC survivors may have complex healthcare needs requiring care from both specialists and primary care. Our objective was to understand how CRC survivors perceive their survivorship care, especially management of their cardiovascular-related chronic diseases.

**Methods:**

We identified patients diagnosed with non-metastatic CRC between 10/1/2007 and 12/31/2015 at Veterans Affairs Medical Centers in North Carolina or Virginia. In 2016, we conducted telephone-based, semi-structured interviews to assess survivors’ experiences with cancer survivorship and changes in health priorities. Interviews were conducted until thematic saturation was reached. Interviews were audio-recorded, transcribed, and coded.

**Results:**

The 25 participants were, on average, 64 years old and approximately 4 years post-CRC diagnosis at the time of interview; most were white (60%), male (92%), and diagnosed with colon cancer (64%) as opposed to rectal cancer. CRC survivors reported: (1) a shift in focus from surviving cancer to reducing cardiovascular disease risk (e.g., by managing weight); (2) challenges with taking medications for CVD-related conditions; (3) new recognition of the importance of engaging with primary care providers.

**Conclusions:**

Experiences with cancer shapes how survivors view their health. Management of cardiovascular-related chronic disease is important to veteran CRC survivors. There is a need to deliver cardiovascular disease risk reduction programs tailored for CRC survivors.

**Electronic supplementary material:**

The online version of this article (10.1186/s12913-018-2975-3) contains supplementary material, which is available to authorized users.

## Background

The Veterans Affairs (VA) healthcare system is the largest integrated healthcare systems in the US and a high-volume provider of cancer care in the US [1]. Among VA patients and across the US, colorectal cancer (CRC) is the third most commonly-diagnosed cancer [1, 2]. Due to robust national programs, approximately 80% of VA patients undergo CRC screening [3, 4]. With earlier detection and advances in cancer treatment, CRC-related death rates for older adults (aged ≥ 50 years) are declining. Currently there are approximately 1 million U.S. CRC survivors [5, 6].

Among cancer survivors, approximately half of non-cancer deaths are attributed to cardiovascular disease (CVD) [7, 8]. Compared to people without a history of CRC, CRC survivors may have moderately increased risk of CVD for several reasons. First, CRC and CVD have similar risk factors (e.g., sedentary behaviors, poor quality diet, short sleep, tobacco use; Table 1; Additional file 1: Figure S1) [9–12]. Secondly, adjuvant treatment for CRC increases the likelihood of developing hypertension, which is a leading CVD risk factor [13]. Finally, for some CRC survivors, the side effects of CRC treatment (e.g., peripheral neuropathy and having an ostomy) [14–16] may make it more difficult to maintain appropriate levels of physical activity [14, 15].

**Table 1:** Factors influencing CRC and CVD

Risk Factors	CRC	CVD
Cigarette smoking	✓	✓
Poor quality diet	✓	✓
Physical inactivity	✓	✓
Short sleep	✓	✓
Diabetes diagnosis	✓	✓
Obesity	✓	✓
Heavy alcohol use	✓	✓
Hereditary conditions (e.g., familial adenomatous polyposis, Lynch Syndrome)	✓	
Elevated lipids/ cholesterol		✓
Family history of CVD		✓
Protective Factors		
Primary care use	✓	✓
Statin use	✓	✓
Aspirin use	✓	✓
Physical activity	✓	✓
Healthy diet	✓	✓

The VA has a rich history of developing chronic disease prevention and CVD risk management programs. These programs provide an integrated approach to controlling chronic diseases through patient education, monitoring, and coordinating services. The high quality of CRC care provided in the VA is well-reputed [17–22]. Yet, relatively little effort has been made to implement chronic disease management specifically in the context of VA cancer survivorship. Similarly, there has been little research addressing CRC survivors’ perceived follow-up care related to CVD risk management [23–25]. Our objective was to understand CRC survivors’ perspectives on cancer survivorship experiences and chronic disease management in VA Medical Centers (VAMCs). We were particularly interested in describing how VA CRC survivors’ health priorities change as they complete active treatment and focus on cancer surveillance and survivorship care. Understanding CRC survivors’ perceptions is an important foundation to develop patient-centered chronic disease management programs tailored to meet the needs of CRC survivors.

## Methods

We identified potential participants from the VA Cancer Cube maintained by the Veterans Health Affairs Support Service Center. The VA Cancer Cube uses the national cancer case ascertainment system, based on histology and first course of treatment, to identify patients with suspected cancers [26].

To be eligible for this study, participants must have been diagnosed with incident Stage I, II, or III cancer of the colon or rectum between October 1, 2007 and December 31, 2015 at VAMCs in North Carolina or Virginia and have a valid home mailing address. We interviewed CRC survivors approximately 1 to 9 years post-CRC diagnosis, a timeframe consistent with previous work [27]. To confirm that patients met the eligibility criteria, we reviewed the electronic health record. Potential participants were mailed a letter explaining the study, a prepaid mailing envelope, and a self-administered survey assessing demographics, symptoms, barriers to care, and adherence with recommended care (e.g., CRC surveillance, medications). On the cover page of the survey, patients indicated whether they agreed to be contacted for a future telephone-based interview. These interviews were the primary data source for this analysis. The VA CRC population and our study sample are primarily comprised of men [1]. To ensure representation of females and minorities, we interviewed all women who provided consent and prioritized interviewing racial minority patients. We then selected participants to ensure equal representation by stage of disease (e.g., I, II, or III) and cancer type. Because their treatment patterns are different (i.e., rectal patients may receive radiation therapy, whereas colon cancer patients generally do not) which affects their side effects, we oversampled rectal cancer patients.

Semi-structured, telephone-based interviews were conducted between May 5, 2016 and August 8, 2016 by two health services researchers trained in behavioral sciences and qualitative interview techniques (SMA and LLZ). Our interview guide incorporated feedback obtained through meetings with primary care professionals, gastroenterologists, social psychologists, and qualitative health services researchers located at the Durham VAMC and Duke University. The questions addressed prioritization of health needs, among other issues. The interview guide questions are summarized in the Additional file 2. In this analysis, we report on survivors’ experiences with cancer survivorship, survivors’ discussion about their prioritization of health needs, and how these have changed since their CRC diagnosis and treatment.

To characterize the sample, we obtained demographic and clinical characteristics from the VA electronic health record. Interviews were audio-recorded using Sparky USB devices and later transcribed (Weston, CT). Qualitative software, ATLAS.ti v6.2 (Berlin, Germany), was used to manage data. We used conventional content analysis to describe the phenomenon of health priority perspective of VA CRC survivors [28]. Codes were derived from review of the interview transcript text rather than being established a priori. Four members of the research team (SMA, LLZ, KMG, and CIV) met to review the first four transcripts to identify an initial set of emergent codes. Once codes had been identified, subsequent interviews were independently coded by two members of the research team (LLZ and KMG). Team members met to discuss questions about coding and reach consensus. The team members then aggregated codes into content categories. Analysts worked together to synthesize results into key themes. This study was approved by the Durham VA Health Care System Institutional Review Board.

## Results

### Participant characteristics

**Fig. 1:**
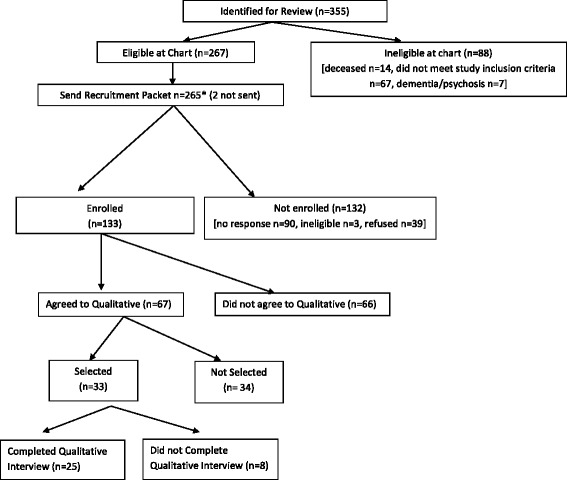
Patient flow

Of the 129 survivors who completed the survey, 66 agreed to be contacted for a qualitative interview. We contacted 33 potential participants, of whom 25 completed the individual interviews (Fig. 1). On average, participants were 64 years old; most were white, married or partnered, males diagnosed with colon (as opposed to rectal) cancer (Table 2). Participants were an average of approximately 4 years post-CRC diagnosis at the time of their interview. Interviews lasted an average of 26 min.

**Table 2:** Characteristics of interview participants (*n* = 25)

	Percent/ Mean	SD (Min, Max)
Age, years	64	6.93 (50, 80)
Sex		
Male	92%	–
Female	8%	–
Race		
White race	60%	–
Black race	28%	–
Other race	12%	–
Marital status		
Married or living with partner	52%	–
Divorced or separated	32%	–
Widowed	4%	–
Single, never married	12%	
Stage at Diagnosis		
Stage 1	32%	–
Stage 2	28%	–
Stage 3	40%	–
Cancer Type		
Colon	64%	–
Rectum	28%	–
Rectosigmoid junction	8%	

Note: These data were derived from patients’ electronic health record

### Shifting focus from surviving cancer to reducing CVD

CRC survivors reported that their focus has shifted from surviving cancer to managing chronic conditions. Some survivors reported that their most significant health priority was now heart health. A man with stage 1 colon cancer reported: “*My biggest problem is my heart… I figure if I take care of my heart, I will be around a whole lot longer*” and “*I was careful for about a year. Then it just went all to pieces. When I started back at the VA, I had talks with the doctors...Then I started changing my living habits drastically.*” Survivors also described a newly found interest in receiving preventive health services, such as annual influenza vaccinations, after their experience with CRC.

Survivors described comorbid conditions that were diagnosed both before and after their CRC treatment; most commonly, CVD-related chronic conditions and arthritis which are responsive to lifestyle behavior change. A man with stage 2 rectal cancer said: “*I had Hepatitis C before I found out I had cancer. And then the diabetes happened after the cancer… But I’m mostly controlling it with my diet and then I was trying to walk a mile a day.*”

Weight management concerns were commonly-expressed by participants as a mechanism to reduce their CVD risk. Some survivors reported low levels of physical activity and poor diet both before and after their cancer diagnosis and treatment, which caused them to gain weight. Conversely, some survivors reported weight loss associated with CRC, which resulted in easier control of their chronic conditions. As an example, a man with stage 1 colon cancer said: *“I’m no longer on insulin… I’m barely a borderline diabetic right now. A lot of that was weight loss. Before I started treatments and everything, I weighed in at 235 pounds. And now I’m keeping my weight down to 180, 185 somewhere around there.”* Survivors also discussed how the cancer experience has shaped their understanding of the importance of weight management. A man with stage 2 colon cancer said: “*I’ve always been more on the plumper side than the thinner side… As a result, I’m becoming more proactive, and that I would say was probably somewhat because of [finishing] my [cancer] treatments. Things that have taken place that have made me more aware of how I can benefit myself by being more astute about what I do”*.

### Challenges with taking medications for CVD-related conditions

CRC survivors reported that their perspectives on medication use had changed over the course of their cancer journey. Survivors now reported taking medications as prescribed to be important to them, whereas before their CRC experience it seemed more of a nuisance. A man with stage 3 colon cancer said: *“[Now I’m] takin’ the medications I’m supposed to…Nothing to do with the cancer. Just the heart stuff.”* Similarly, a second man with stage 1 colon cancer reported: “*I used to have medications and I wouldn’t take [them] like I was supposed to, but now I make sure that that I do.*”

Survivors also reported that their medication needs have changed after cancer treatment. A man with stage 3 colon cancer and comorbid diabetes stated: *“I take insulin every night… [When] you take chemo, you know, [your blood sugar] automatically shoots up 200 … [After the] treatment, my sugar levels are different now… I was just taking pills for my sugar diabetes, now I’m taking 30 units of insulin every night. I hope that [it doesn’t get] worse… It’s all different…”.*

Despite the uniqueness of the cancer experience in motivating better medication adherence, CRC survivors reported barriers that are similar to the general population. [29, 30] These included problems with polypharmacy (e.g., taking multiple medications daily), scheduling (e.g., difficulty taking medications at specific times of day), forgetfulness, lacking support, being worried about taking them for the duration of life, struggling with depression, and running out of a prescription too soon. A man with stage 2 colon cancer described his medication behaviors this way: “*I just know that I take all my medicines, on time, every day. My wife sees to that because of this memory loss I have….*”

### New recognition of the important of engaging with primary care providers

CRC survivors reported working with their VA primary care provider for chronic disease management, including medications. When connected with primary care, survivors noted that their primary care provider was charting the course for post-cancer care and providing traditional chronic disease management. A man with stage 3 colon cancer reported: “*I visited with [my primary care provider] several times and she laid out my plan… I have high blood pressure and I take medication for that. [She] makes sure I’m following up and checking my blood pressure and [that I] have my prescription pills... Those are things my VA primary care doctor does for me. When I need a refill it’s just a matter of dialing a number and refilling my prescription no question about it.”*

### Findings consistent with existing literature

The CRC survivors in our cohort confirmed experiences reported in other literature, thus we did not elevate these finding to the level of an emerging theme [31, 32]. In addition to the key themes that we identified, survivors also reported changes in their daily routines. A man with stage 2 rectal cancer stated: “*You learn to keep up with your bowel movements… You know some days you can get by with doing more than you can on other days [and] plan things accordingly*.” Despite these changes, survivors expressed resiliency and emphasized the importance of self-care in returning to a “new normal.” While this idea of changing daily routines and a “new normal” are important, it has been well described in existing literature and thus we did not identify it as an emerging theme in this analysis [33, 34].

Cancer survivors may have more complex care coordination needs that are time-dependent. During survivorship care, coordination of health care services may become a priority. They also reported care coordination advantages of receiving all their care within an integrated healthcare system. A man with stage 3 colon cancer reported: “*My doctors there at [the VA] are an outstanding crew… If I run into any medical problems I try to maintain the same doctors who have a history of my medical needs who can better assist me to live on a day to day basis...*” Similarly, another man with stage 3 colon cancer said: “*They all communicated very well between each other because of the [integrated electronic health] record. I’d go see my primary care doctor and he’d have all my results from the CAT scans, all my blood work information, he always had everything right at his fingertips. They all did*.”

## Discussion

We sought to understand how CRC survivors perceive their survivorship care related to CVD. We identified several new themes (e.g., shifted from surviving cancer to managing chronic conditions, challenges with taking chronic medications; recognition of the importance of engaging primary care providers) as well as several themes consistent with the general patient population and other cancer survivors cohorts (e.g., changes in daily routine, issues with care coordination) [14, 15, 20, 35–37]. In addition, CRC survivors reported CRC treatment side effects, such as peripheral neuropathy and changes in bowel habits, that would require adapting CVD risk reducing interventions due to a need to modify behavioral lifestyle recommendations.

### Maintaining physical activity and weight management

Physical activity is associated with improved survival among CRC survivors. For example, engaging in leisure-time physical activity is associated with lower all-cause mortality [9]. Thus, engaging in physical activity is important. Clinicians could ask CRC survivors about symptoms impacting their willingness to engage in physical activity, and create alternative suggestions for ways to remain active (e.g., walking on smooth surfaces, taking an extra lap around the grocery store to add activity into daily routines).

Physical inactivity is associated with weight management problems and CRC survivors who are overweight are more likely to suffer from comorbid CVD [38]. CRC survivors in our cohort reported weight loss coinciding with their CRC diagnosis, which is associated with worse outcomes and increased mortality [39]. This is part of the “obesity paradox” and appropriate weight management among cancer survivors is remains important [40]. CRC survivors reported that their bowel habits changed, prompting them to change their dietary patterns. Clinicians should address weight management with their CRC survivors, asking whether side effects of treatment are making weight management challenging.

### Taking medications as prescribed

Survivors reported barriers similar to those reported by patients with chronic conditions (e.g., forgetting to take their medications, not getting them refilled in time) [30]. Evidence suggests that nonadherence may be more common among cancer survivors than the general population and that nonadherence may vary based on cancer treatment and other characteristics. A recent study evaluated adherence to cardiovascular related medications (e.g., statins and ACEIs/ARBs/β-blockers) in the 12 months before and 12 months after cancer diagnosis among elderly Medicare beneficiaries with colorectal, prostate, and breast cancers [41]. The authors found that adherence to both statins and ACEIs/ARBs/β-blockers was only about 31% during the 4 months following patients’ cancer diagnosis. An important minority, approximately 14% of patients, were not adherent to both medication classes (e.g., statins and ACEIs/ARBs/β-blockers) [41]. Even in an integrated healthcare system, adherence is problematic for cancer survivors. Another study conducted among early stage breast cancer survivors receiving care in an integrated healthcare system found that 75% of patients were nonadherent [42]. Whether these challenges translate into different medication adherence behaviors has not been well studied. Thus, medication adherence for chronic disease medications may be particularly important and life-prolonging for CRC survivors.

Medical oncologists and primary care providers have different perceptions regarding cancer survivorship activities [43] and differing perspectives on models of cancer survivorship care [44]. Primary care may be particularly important partner to reduce CVD risk among CRC survivors. CRC survivors report holding the advice of their providers in high esteem [45] and primary care use is associated with lower CRC incidence and reduced all-cause mortality [46].

### Study limitations

Our analysis involves CRC survivors receiving VA healthcare in the southeastern United States. In an effort to include a wide array of perspectives, we interviewed survivors with both colon and rectal cancer and who were 1 to 9 years post-diagnosis; thus, our findings may not be generalizable to experiences among survivors who were closer or farther from their time of diagnosis. Our findings regarding challenges with taking medications do not consider how many medications participants were prescribed. While the qualitative interview approach allows for better understanding of the patient perspective and provides rich information, it does not elucidate survivors’ behaviors with objective data (e.g., medication adherence, physical activity). Other study designs, such as prospective observational studies, may be employed to characterize CVD-related health behaviors among CRC survivors.

## Conclusions

It is important to note that many of the survivors in our study identified a specific CVD risk-reducing behavior (e.g., weight management, engaging in physical activity) that was of increased importance to them after their cancer experience. However, evidence suggests that improvements in mortality and recurrence risk may not be attributed to a single behavior but instead to a holistic healthy lifestyle [47]. There is a need to deliver CVD risk management programs that address multiple risk reduction strategies (e.g., diet, exercise, sleep, medication adherence) and which are tailored for CRC survivors. CVD risk management programs for cancer survivors should acknowledge the cancer experience, make recommendations tailored for survivors’ abilities (e.g., physical activity recommendations adapted for survivors with peripheral neuropathy; diet recommendations adapted for survivors managing changed bowel movement frequency; fatigue), and include education about cancer surveillance. These programs should educate patients jointly on the importance of engaging in healthy behaviors to reduce their likelihood of CRC recurrence, reduce their CVD risk, and improve their quality of life. Primary care providers, in conjunction with their oncology colleagues, may be uniquely positioned to support this effort.

## Additional files

Additional file 1: **Figure S1.** Association between CRC and CVD risk factors. This figure depicts individual and shared risk factors for CRC and CVD. (DOCX 266 kb)

Additional file 2: Qualitative guide sample interview questions. This is an example of questions asked during individual qualitative interviews with colorectal cancer survivors. (DOCX 63 kb)

## Abbreviations

CRC: Colorectal cancer
CVD: Cardiovascular disease
US: United States of America
VA: Veterans affairs
VAMC: Veterans Affairs Medical Center
