# Supplementary figures and images for: Chronic disease management perspectives of colorectal cancer survivors using the Veterans Affairs healthcare system: a qualitative analysis
Source: BMC Health Serv Res. 2018 Mar 9;18:171. doi: 10.1186/s12913-018-2975-3 (PMC5845139; doi:10.1186/s12913-018-2975-3)

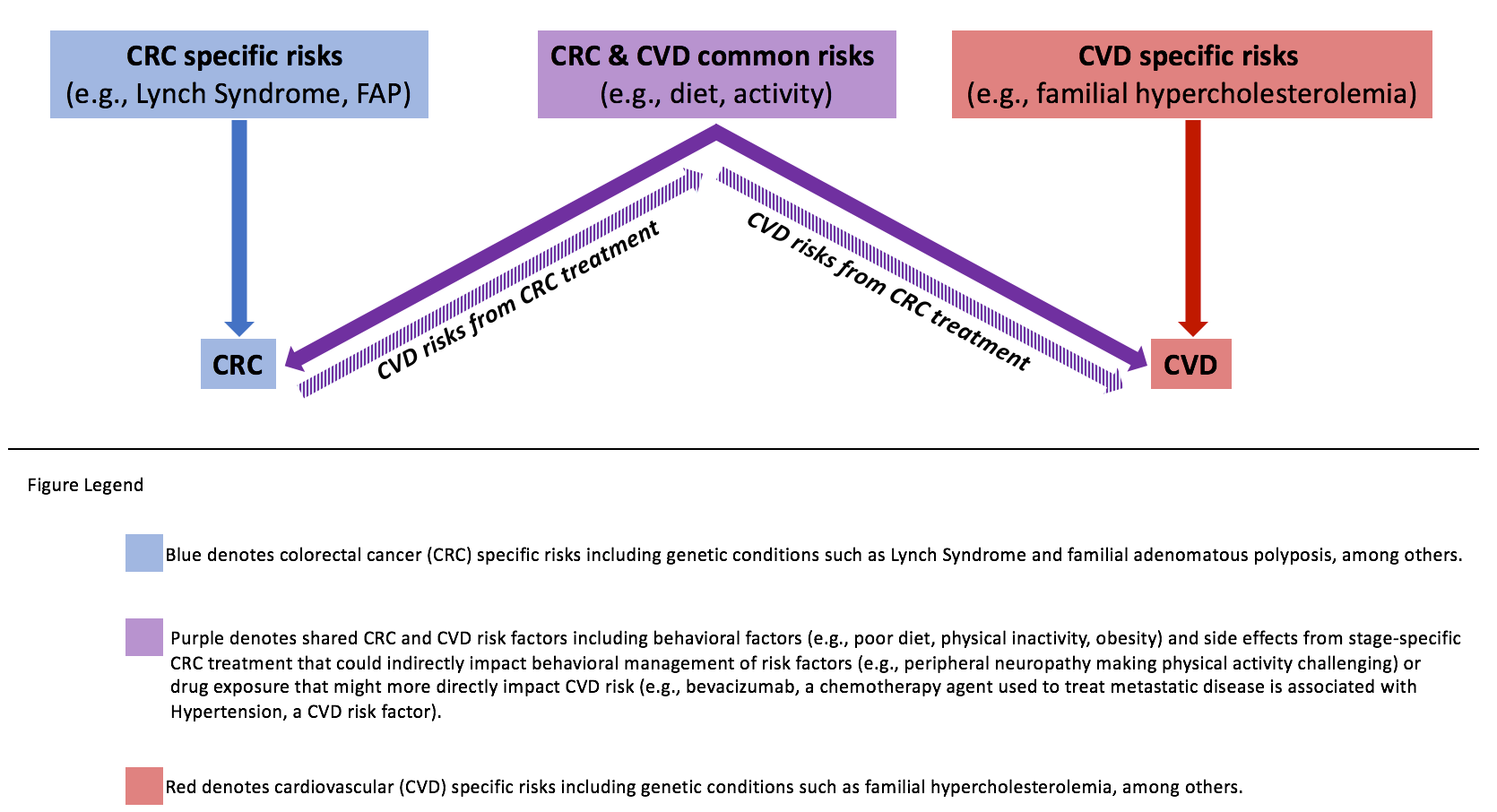

Supplement: Supplementary file 1 — Figure S1. Association between CRC and CVD risk factors. This figure depicts individual and shared risk factors for CRC and CVD. (DOCX 266 kb) [file 12913_2018_2975_MOESM1_ESM.docx]
