# Supplementary material for: Chronic disease management perspectives of colorectal cancer survivors using the Veterans Affairs healthcare system: a qualitative analysis
Source: BMC Health Serv Res. 2018 Mar 9;18:171. doi: 10.1186/s12913-018-2975-3 (PMC5845139; doi:10.1186/s12913-018-2975-3)
Supplement: Supplementary file 2 — Qualitative guide sample interview questions. This is an example of questions asked during individual qualitative interviews with colorectal cancer survivors. (DOCX 63 kb) [file 12913_2018_2975_MOESM2_ESM.docx]

**Care Transitions Domain: Sample Questions**

- When you completed your cancer treatment, who communicated with you about what should happen next?
- What were you told about what would happen next?
- Looking back, what additional information do you wish you had known when you finished your colorectal cancer treatment?

**Prioritization of Health Needs Domain: Sample Questions**

- How do you think about prioritizing your different health conditions now that you have had cancer?
  - Prompt: What has changed?
- What kinds of things are important to you now about your health?
- What are some things that you struggle with related to you health or healthcare?
  - Prompt: Taking medication? Getting to the VA? Knowing who to call when you have questions?
- What kind of information would be helpful to other people in your shoes?
